# Electromagnetic Field Stimulation Attenuates Phasic Nociception after Complete Spinal Cord Injury in Rats

**DOI:** 10.3390/brainsci11111431

**Published:** 2021-10-28

**Authors:** Suneel Kumar, Ajay Pal, Suman Jain, Thirumurthy Velpandian, Rashmi Mathur

**Affiliations:** 1 Department of Physiology, All India Institute of Medical Sciences, New Delhi 110029, India; 80ajay@gmail.com (A.P.); sumanjain10@gmail.com (S.J.); mathurashmi@yahoo.co.in (R.M.); 2 Department of Ocular Pharmacy and Pharmacology, All India Institute of Medical Sciences, New Delhi 110029, India; tvelpandian@hotmail.com

**Keywords:** complete spinal cord injury, electromagnetic stimulation, hyperalgesia and allodynia, glutamate, H-reflex, nociceptive flexion reflex, glial cells, neurotransmitter

## Abstract

Traumatic spinal cord injury (SCI) is one of the most incapacitating pathologies, leading to huge rehabilitation challenges besides a social-economic burden on SCI patients and their families. There is no complete curative treatment available so far. Non-invasive and patient-friendly use of extremely low-frequency electromagnetic field stimulation (EMF) has emerged as a therapeutic and rehabilitation option. In this study, we tested whole-body EMF stimulation on thoracic complete SCI-induced nociception including sensorimotor deficits in rats. The EMF application significantly attenuated hyperalgesia and allodynia to thermal, electrical, and chemical stimuli from 6 weeks onwards as well as restoration of spinal reflexes, viz., H-reflex and nociceptive flexion reflex at the study endpoint (week 8). Besides, massively increased glutamate at the SCI injury site was observed in SCI rats with no treatment, which was also attenuated significantly by EMF stimulation. Spinal cord histology of the injury area showed a decrease in lesion volume and glial population in the EMF-stimulated rats. These findings indicate the beneficial role of EMF stimulation after thoracic complete SCI in adult male rats and, thereby, a beneficial patient-friendly rehabilitation tool.

## 1. Introduction

Traumatic spinal cord injury (SCI) is a direct insult to the spinal cord that causes functional impairment via direct or indirect trauma. These processes occur instantly after an insult and, then, are consistent for several weeks after SCI, irrespective of injury mode and severity [1]. This creates abnormal activity in spinal and supra-spinal generators, which further leads to hypersensitivity of dorsal horn sensory neurons [2]. This hypersensitivity is contributed by an increase in the neurons responding to noxious stimuli, spontaneous background activity, evoked activity to formerly innocuous and noxious stimuli, alterations in sodium currents, activation of microglia [3], and alteration in excitatory amino acids such as glutamate [4].

There is no treatment for SCI patient’s complete recovery after this neurotrauma. These patients suffer continuously from ongoing pain and immobility that leads to other secondary complications. However, magnetic, or electromagnetic field (EMF), stimulation has shown the potential to activate regenerative processes in vitro and in vivo [5]. It is been reported to improve locomotion, restore muscle contraction and their related properties, inhibit inflammation and oxidative stress [6], and limit muscle degeneration besides sparing white matter and smaller lesion volume in different animals mild to moderate injury SCI models except for complete transection of the spinal cord and SCI patients’ trials [1,7]. Our lab reported the beneficial effects of EMF on the improvement of feeding behavior [8], locomotion [9], formalin tonic pain, and brain neurotransmitters, while reducing osteoporosis activity, lesion volume, and free radicals [5,10,11] after complete SCI. In hemisection SCI, EMF stimulation improves electrophysiological functions such as H-reflex [12] in a rat model. However, there is no report in an experimental setting on the use of EMF stimulation on attenuating neuropathic pain including allodynia and hyperalgesia after complete transection of the spinal cord.

Therefore, we have designed this study to investigate the role of chronic EMF stimulation on behavioral, electrophysiological, and neurochemical correlates of allodynia and hyperalgesia after complete SCI in adult male rats.

## 2. Materials and Methods

Adult male Wistar rats (200–250 g, *n* = 55) were used in this study. All experiments were done following the Institute Animal Ethics Committee (IAEC, No. 308/IAEC/05) of the All India Institute of Medical Sciences (AIIMS), New Delhi, India. They were bred and reared in the AIIMS Central Experiment Facility. They were housed in polypropylene cages (50 cm × 20 cm × 15 cm). Laboratory food pellets (Ashirwad Industries, Ropar, Haryana, India) and clean fresh water were provided ad libitum to them. One animal in each cage was kept post-SCI to minimize the spread of infection. Cages were cleaned and changed weekly including standard bedding. They were maintained and housed under controlled room temperature (24 ± 2 °C) and a light–dark cycle (14:10 h).

### 2.1. Complete Spinal Cord Injury and Postoperative Care

Rats were deeply anesthetized using a mixture of ketamine and xylazine (60 mg/kg and 10 mg/kg, respectively). Rats have undergone laminectomy (Sham) followed by complete thoracic (T11) transection of the spinal cord. Briefly, the spinal cord was exposed after bilateral laminectomy of T10–T12 vertebrae and was transected completely by fine dura micro scissor at the T11 vertebra. The severed ends of the spinal cord typically retracted about 1–2 mm and, then, gently lifted to ensure completeness of spinal cord transection. The gap was, then, filled with gel foam (AbGel, New delhi, India), and skin and underlying tissues were sutured layer by layer. During and after surgery, body temperature was maintained using controlled heating pads (CMA-150, MA, USA). Postoperative care was carried out as per standard lab protocol including the rats’ re-hydration with a bolus of Lactate Ringer’s solution (5 mL, I.P.), and they were treated with a wide spectrum of antibiotics (gentamycin, 15–20 mg/kg, 3–5 days; cefazoline, 50–100 mg/kg, 5–7 days; i.m.). Antibiotic powder (Neosporin, GlaxoSmithKline, India) was applied locally at the incision site. The rats also received manual bladder expression daily until reflexive bladder control returned. The complete SCI was confirmed 24 h later by recording nadir BBB score and MRI imaging (Figure S1, *n* = 3), and then, half of the rats were exposed to EMF stimulation in a specifically designed chamber. A few rats died (9-SCI and 2-EMF) due to post-SCI complications, and the remaining rats completed the study (Figure S2, *n* = 41; Sham = 15, SCI = 12, and EMF = 14).

### 2.2. EMF Stimulation Chamber

Twenty-four hours following SCI/sham injury, rats in the EMF group were stimulated to an EMF of 17.96 μT (50 Hz) for 2 h daily for 8 weeks in a specifically designed chamber as per standard published protocol [9,10,11]. Briefly, it consisted of 2 inner and 2 outer electromagnetic coils with 8 and 18 turns, respectively. The diameter of the coils was 1000 mm with a 45 mm width. The distance of the center of outer coils from the center of the structure was 470 mm, while that of inner coils was 122 mm. The current in the electromagnetic coil was 1 amp, and EMF was 17.96 μT with 50 Hz in the center where the rats were placed on the stand. The rat cage had separate compartments for 6 rats (1 rat/compartment). The intensity of EMF was monitored every day before and after the exposure by utilizing a magnetometer (Walker Scientific Inc. Auburn Hills, MI, USA). The SCI group of rats was stimulated to EMF similarly, except the coils were not activated by the power supply.

### 2.3. Behavioral Assessment

All the rats were assessed for sensorimotor functions including motor recovery and hyperalgesia/allodynia development (Sham = 15, SCI = 12, and EMF = 14). To fulfill the aim of the study, we tested all the rats with thermal, electrical, and chemical stimuli using a battery of sensorimotor tests. Sensorimotor responses were recorded below the injury from affected tails and paws of rats before and after SCI weekly (Figure S3).

#### 2.3.1. BBB Score

BBB score was assessed weekly pre- and post-injury via the 22-point Basso, Beattie, and Bresnahan (BBB) graded locomotor rating scale (0—hindlimbs complete paralysis and 21—normal locomotion) method for functional recovery [13].

#### 2.3.2. Tail Flick Latency (TFL)

Sensorimotor responses of the tail were recorded to various thermal (cold/hot) stimuli as TFL. It was noted after immersion of tail (4 cm from base) into an inner glass reservoir of Dale’s bath (INCO, India) filled with water of varying temperatures (5 ± 0.5 °C to 50 ± 0.5 °C). The water of the inner bath was altered after 30 min from cold (5 °C and 10 °C) to hot (45 °C and 50 °C). A cut-off of 60s for 5 °C, 10 °C, and 45 °C, while 15 s for 50 °C stimuli was pre-set to circumvent injury to the tail [14].

#### 2.3.3. Threshold of Tail Flick (TTF)

It is a segmental motor response, which is mediated via the spinal cord. In this test, nociceptive afferents of the tail were stimulated by noxious electrical stimulation (biphasic square wave pulse of 40 Hz frequency, 1.5 ms duration, and varying current strength of 200 μV), and the threshold to tail flick was recorded and compared between the studied groups [14].

#### 2.3.4. Hind Paw Withdrawal Latency (HPL)

Sensorimotor responses of paws were recorded to thermal noxious stimuli, and withdrawal responses were recorded as HPL. The rat was placed on a pre-heated hot plate set at 52 ± 0.5 °C, while the cut-off time was set at 30 s. Simultaneous with the placement of the rat, the timer was started by a pedal switch. The time elapsed between the placement of the rat to the withdrawal of hind paw (s), became frozen on the monitor, and was recorded automatically as HPL [14].

#### 2.3.5. Acetone Test

The sensorimotor responses of paws to noxious cold chemical stimuli were tested by a drop of acetone (100 μL, 80%) from a micropipette on the plantar surface of the hind paw. The responses were categorized as: 0—no response; 1—startle; 2—withdrawal of paw; 3—withdrawal of paw/flinching/licking of paw/vocalization; 4—category 3 persisting for >10 s [14].

After completing the behavioral assessment, half of the rats from each group were used for electrophysiological assessment and neurotransmitter studies, while the leftover rats were used for histological studies as mentioned under each figure legend.

### 2.4. Electrophysiological Assessment

Electrophysiological indicators of neuronal excitability were studied by Hoffmann reflex (H-reflex) and nociceptive flexion reflex (NFR). These responses were recorded at the end of 8 weeks in anesthetized rats, and the rats’ respiratory rates were monitored continuously during the experiment.

#### 2.4.1. H-Reflex

To record this response, a pair of needle electrodes were placed on the tibial nerve for stimulation (0.1 ms pulses, cathode proximal on nerve), and another pair of needle electrodes were inserted in the digital interosseous muscles between the 4th and 5th metatarsals [12,14]. A ground electrode was inserted subcutaneously into the skin of the tail. The direct muscle response (M-response) and the monosynaptic reflex response (H-reflex), thus, obtained were displayed on the cathode ray oscilloscope (CRO). Muscle response was evoked by single stimuli of increasing intensity (0.16 Hz, 0.2 mA–5.0 mA) until they reached maximal amplitude. The intensity of the stimuli was further increased (supramaximal stimuli) to ensure that all motor or large sensory axons of the nerve were stimulated. Sixteen responses were averaged online. The threshold, latency, and amplitude were measured and compared between groups using software (D-147, DATAQ, USA).

#### 2.4.2. Nociceptive Flexion Reflex (NFR)

After 48 h, the same rats were used to record the NFR under anesthetized conditions [14]. The posterior biceps’ femoris muscle was exposed on one side, and a pair of needle electrodes were inserted for recording. The flexion reflex was elicited by subcutaneous electrical stimulation applied to the hind paw innervated by the sural nerve through a pair of needle electrodes using an increasing intensity of rectangular pulses (2 ms of 0.6–11 mA, 0.16 Hz). Eight responses were averaged online and displayed on CRO similarly. The threshold, latency, duration, and amplitude were measured and compared between groups using software (D-147, DATAQ, Akron, OH, USA).

### 2.5. Neurochemical Assessment

These rats were, then, sacrificed by decapitation and the spinal cord (cervical, thoracic, injured, below injury lumbar, and sacral) segments were dissected under ideal conditions and stored under −80 °C until analysis. These spinal segments were assessed for the excitotoxic glutamate concentration using liquid chromatography mass spectrometry (LC-MS-ESI; API 4000Q Trap-Applied Bio-systems, Foster City, CA, USA) in single ion monitoring mode (SIM) method as described elsewhere [10].

### 2.6. Histological Assessment

To study the histological changes post-SCI and the effect of EMF stimulation, a separate group of rats was sacrificed after the behavioral studies as mentioned above, and rats were, then, perfusions fixed with 4% paraformaldehyde. Longitudinal cryosections (20 μm) including injury area (Microm HM 550, Thermo Scientific, Kalamazoo, MI, USA) were cut, and 5 central sections per rat were stained with cresyl-violet for lesion volume [9,10,11,12] calculation (area x thickness) utilizing software (NIS Elements AR 3.0, city, Japan) and glial cells counting proximal and distal to the injury site [15,16].

### 2.7. Statistical Analysis

The experimental data are presented as mean ± standard error of the mean. The glutamate data were log-transformed first due to heterogeneity before analysis. All the data were analyzed using a one-way analysis of variance followed by Bonferroni post hoc test for multiple comparisons (SPSS 13.0 software, SPSS Inc., IBM, Armonk, NY, USA) as well as student *t*-test when compared to only SCI and EMF groups. A *p*-value  <  0.05 was considered a statistically significant difference.

## 3. Results

### 3.1. Behavioral Assessment

#### 3.1.1. BBB Score

**Figure 1:**
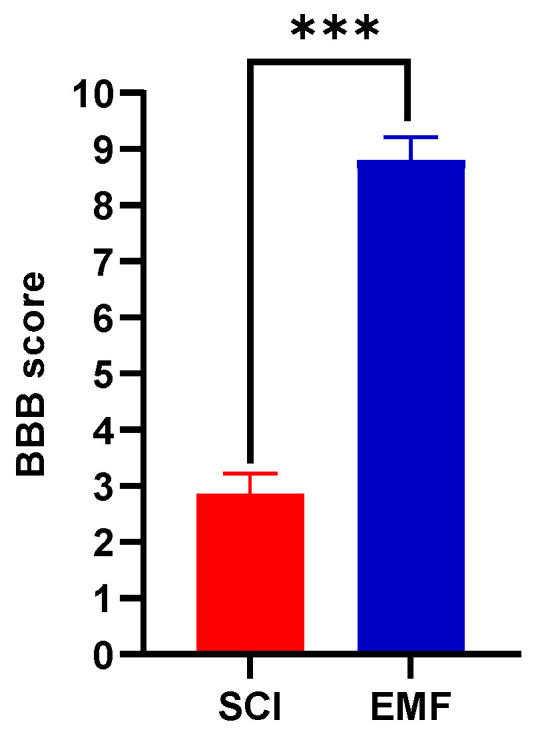
Beneficial protective effect of EMF stimulation after complete SCI on locomotion. The representative BBB score data were compared between SCI (*n* = 12) and EMF (*n* = 14) groups of rats at week 8. *** *p* < 0.001 indicates a comparison between SCI and SCI-treated EMF groups.

Over 8 weeks, rats were studied for their behavioral assessment and their locomotion as per study design (Figure S3). The complete SCI was confirmed using the day 1 nadir BBB score as well as studying the MRI images of the spinal cord after SCI (*n* = 3, Figure S2). We already published the beneficial effect of EMF on locomotor BBB score, which was similarly improved in these rats as well. In the current study, we also assessed BBB score weekly but only presented here the BBB core data at week 8 for simplicity between SCI and EMF groups (Figure 1). The BBB score after 8 weeks was improved gradually in SCI (score 2.85 ± 0.09, joints movement only) and EMF (score 8.8 ± 0.11, sweeping or weight support) groups. When compared between groups, it was significantly higher (*p* < 0.001) in the EMF group as compared to the SCI group.

#### 3.1.2. Sensorimotor Response of Tail to Thermal and Electrical Stimulus

**Figure 2:**
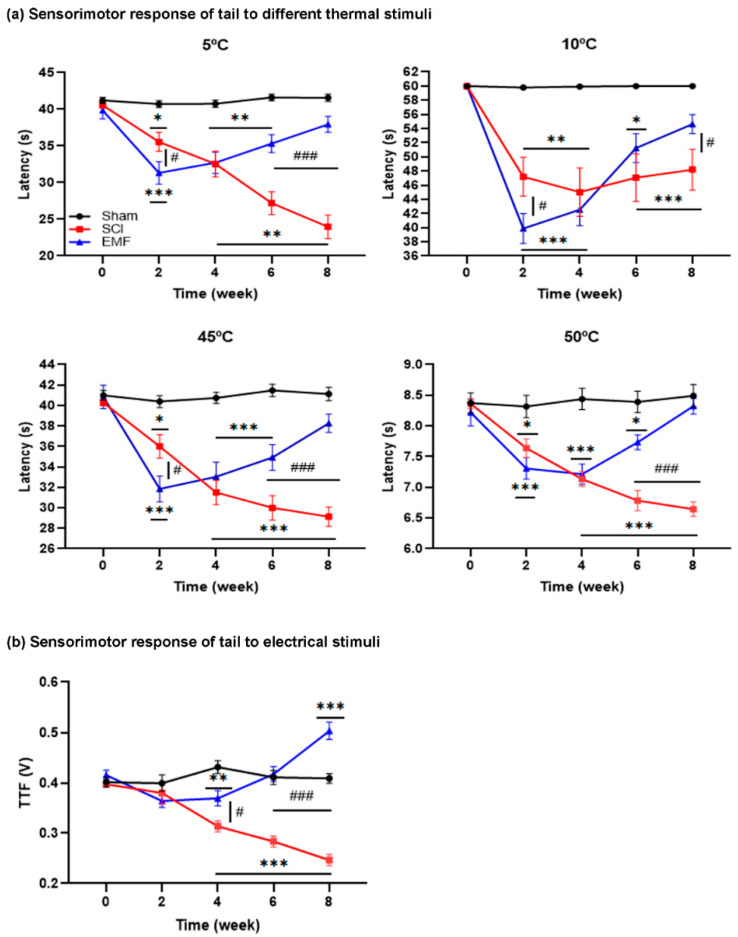
Effect of EMF stimulation on nociceptive responses of the tail to thermal and electric stimuli in SCI rats. Tail flick latencies to different thermal stimuli (**a**) and electrical stimuli (**b**) in the Sham (*n* = 15), SCI (*n* = 12), and EMF (*n* = 14) groups of rats. * indicates a comparison between Sham and SCI/EMF groups, while # indicates a comparison between SCI and EMF groups. */^#^ *p* < 0.05, ** *p* < 0.01, and ***/^###^ *p* < 0.001.

The Sham group did not vary significantly pre- to post-surgery (*p* > 0.05) during the 8 weeks study to any thermal (TFL, Figure 2a) and electrical stimuli (TTF, Figure 2b). As compared to their pre-surgery TFL, the TFL to 5 °C in the SCI group decreased during week 2 (35.56 ± 1.28 s, *p* < 0.012) and progressed until week 8 (23.97 ± 1.61 s, *p* < 0.012), while to 10 °C, the decrement in TFL during week 2 (47.19 ± 2.74 s, *p* < 0.012) was maintained until week 8 (48.19 ± 2.87 s, *p* < 0.012). However, the TFL also decreased in the EMF group until week 4, which gradually improved and was comparable to pre-surgery TFL. In comparison to the Sham group, TFL to 5 °C and 10 °C was lower in the SCI and EMF groups from weeks 2–8 and weeks 2–6, respectively. In the EMF group, TFL was gradually restored from weeks 6–8 and was comparable to Sham at week 8.

In the SCI group, TFL to 45 °C and 50 °C decreased significantly (*p* < 0.012) during week 2 (35.99 ± 1.15 s, 7.64 ± 0.15 s, respectively), which progressed until week 8 (29.13 ± 0.95 s, 6.64 ± 0.11 s, respectively). On the contrary, it did not progress in the EMF group. In comparison to the Sham group, TFL was lower in the SCI group during weeks 2–8 and in the EMF group during weeks 2–6. On the contrary, it started recovering from week 6 and was statistically not significant to the Sham group during week 8.

In the SCI group, TTF progressively decreased from weeks 2–8 compared to week 0; whereas, TTF significantly decreased during weeks 2 and week 4 only in the EMF group. The TTF of SCI versus the Sham group was significantly lower during weeks 4–8. In the EMF versus Sham group, TTF was lower during week 4 only, was comparable during week 6, and then, gradually increased at week 8. TTF was significantly higher in the EMF compared to the SCI group at week 4 and weeks 6–8 (Figure 2b). The overall result suggests severe hyperalgesia after SCI, while continuous stimulation of EMF on SCI rats attenuated hyperalgesia from week 4 onwards and was comparable to the Sham group at weeks 6 and 8.

#### 3.1.3. Sensorimotor Response of Paws to Thermal and Chemical Stimulus

**Figure 3:**
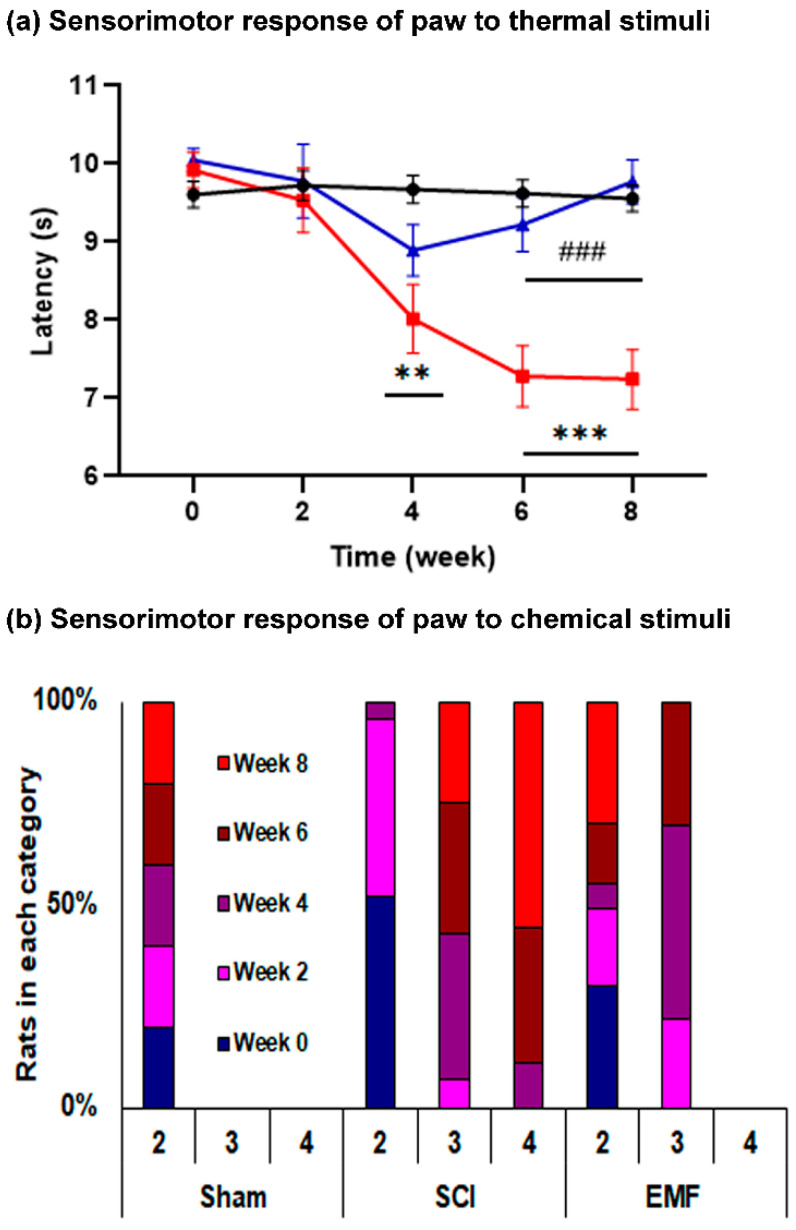
Effect of EMF stimulation on nociceptive responses of paws to thermal (**a**) and chemical stimuli (**b**) in the Sham (black line, *n* = 15), SCI (red line, *n* = 12), and EMF (blue line, *n* = 14) groups of rats. * indicates a comparison between Sham and SCI/EMF groups, while # indicates a comparison between SCI and EMF groups. ** *p* < 0.01 and ***/^###^ *p* < 0.001.

HPL: It progressively decreased (*p* < 0.001) from week 4 (8.02 ± 0.41 s) to week 8 (7.24 ± 0.35 s) in the SCI group versus their pre-surgery HPL (9.92 ± 0.21 s), while it did not vary in the EMF group. In comparison to the Sham group, HPL was lower in the SCI group from week 4 through week 8; whereas, it was restored and comparable to the EMF group (Figure 3a).

Acetone test: The number of rats in category 4 during acetone stimuli gradually increased from week 4 through week 8 in the SCI group, while it was a nadir in the Sham and EMF groups. The number of rats in the EMF compared to the SCI group was less during week 6 and week 8 in category 3 (Figure 3b). The overall result suggests the severe hyperalgesia and allodynia of hind paws after SCI, while continuous stimulation of EMF attenuated hyperalgesia and allodynia from week 4 onwards and was comparable to the Sham group at weeks 6 and 8.

### 3.2. Electrophysiological Assessment

#### 3.2.1. M/H-Reflex

**Figure 4:**
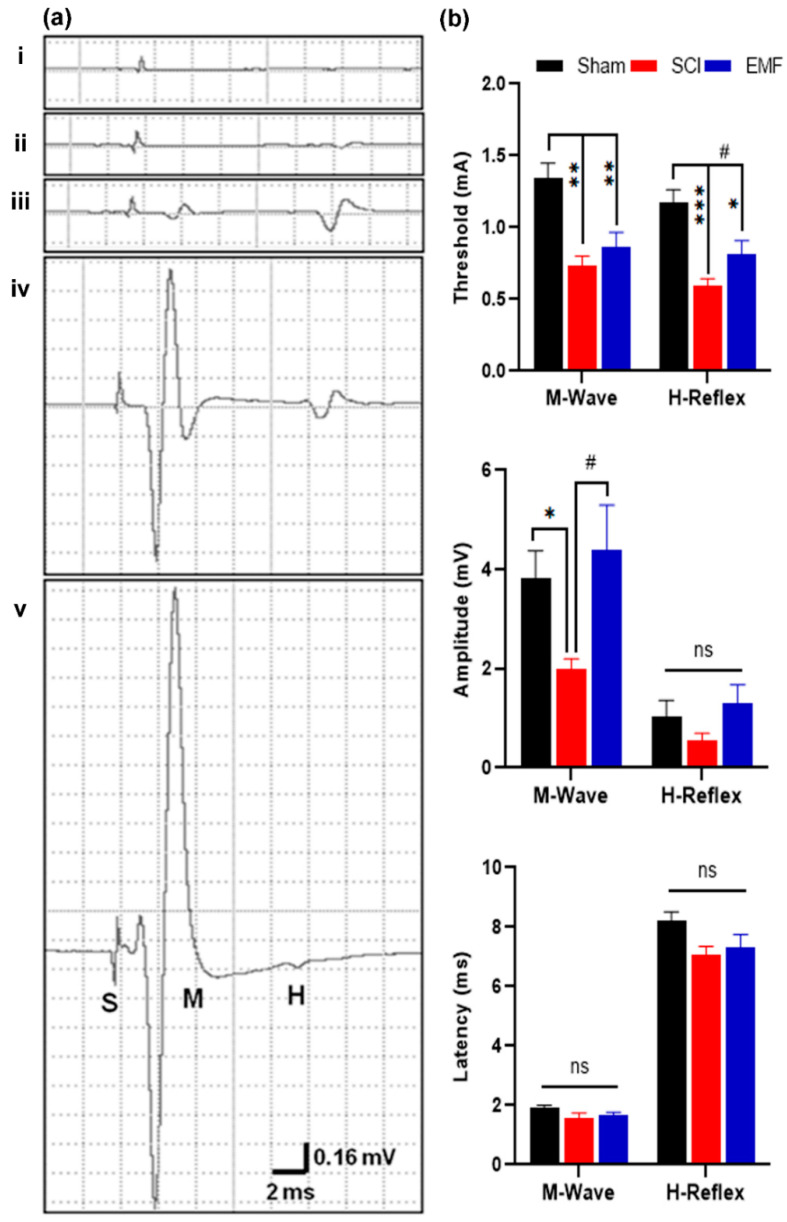
Effect of EMF stimulation on M-wave and H-reflex after complete SCI. It shows the representative graphs of M-response and H-reflex (**a**) (i–v). In the Sham group, graph a-i shows stimulus artifact (S) only to 1.2 mA (sub-threshold stimulus); graph a-ii shows the appearance of H-reflex to 1.4 mA (threshold stimulus); graph a-iii also shows the appearance of M-response to 1.6 mA (threshold stimulus); graph a-iv shows the maximum amplitude of M-response and H-reflex, and graph a-v shows the disappearance of H-reflex only. Panel (**b**) shows the threshold, amplitude, and latency of M-response and H-reflex in the Sham (*n* = 7), SCI (*n* = 6), and EMF (*n* = 6) groups. * indicates the comparison between Sham and SCI/EMF groups, while # indicates the comparison between SCI and EMF groups. */^#^ *p* < 0.05, ** *p* < 0.01, and *** *p* < 0.001.

We also studied M-response and H-reflex responses in these rats. The threshold of M-response and H-reflex was significantly lower in the SCI (*p* < 0.01) and EMF (*p* < 0.001; *p* < 0.05, respectively) groups of rats as compared to the Sham group of rats (Figure 4a,b). It shows neuronal hypersensitivity after 8 weeks of SCI. However, the H-reflex threshold was partially restored by EMF as compared to SCI (*p* < 0.05). The amplitude of M-response was also lower in SCI (*p* < 0.05) compared to Sham; whereas, it was restored by EMF stimulation (*p* > 0.05; *p* < 0.05 versus SCI). H-reflex amplitude was lower in SCI (0.55 ± 0.15 mV) compared to Sham (1.03 ± 0.33 mV), while it was improved (1.30 ± 0.36 mV) by EMF (Figure 4b) stimulation. However, there is no significant difference in latency amongst the groups.

#### 3.2.2. Nociceptive Flexion Reflex

**Figure 5:**
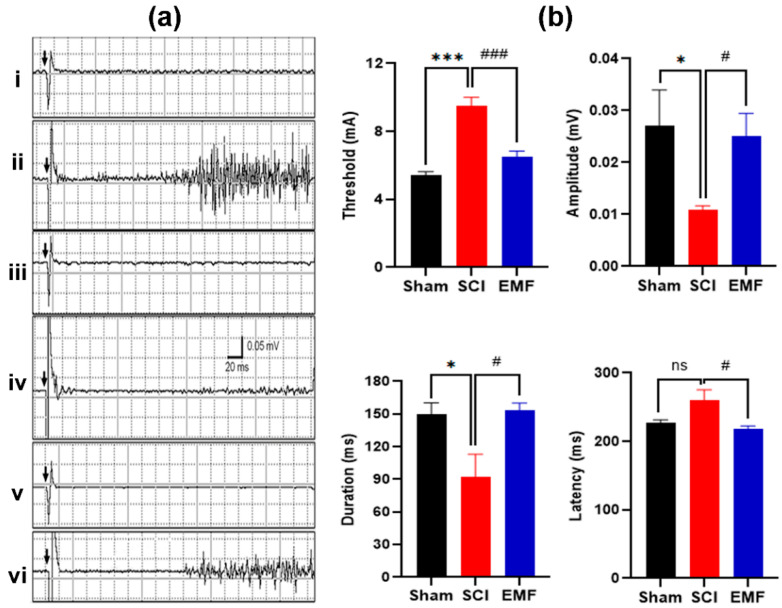
Effect of EMF stimulation on NFR after complete SCI. The representative graphs of NFR (**a**) in the Sham (i–ii, *n* = 7), SCI (iii-iv, *n* = 6), and EMF (v–vi, *n* = 6) groups. Graphs i, iii, and v (4.8, 9.8, 6.8 mA, respectively) show the stimulus artifact (black arrows), while ii, iv, and vi show the threshold for NFR in the Sham (5 mA), SCI (10 mA), and EMF (7 mA) groups. The right-side panel (**b**) shows the comparison of NFR parameters such as threshold, latency, amplitude, and duration. * indicates the comparison between the Sham and SCI/EMF groups, while ^ indicates the comparison between the SCI and EMF groups. */^#^ *p* < 0.05 and ***/^###^ *p* < 0.001.

Similarly, NFR responses were recorded at the end of the study point. NFR is a complex response involving multiple synapses (Figure 5a,b). NFR threshold and latency were higher (*p* < 0.01 and *p* > 0.05, respectively, versus Sham group), while amplitude and duration were lower in the SCI group (*p* < 0.05 versus Sham group); whereas, these parameters were improved significantly by continuous EMF exposure. The threshold of NFR in the EMF group improved (*p* < 0.001) in the latency, amplitude, and duration of NFR as compared to SCI (*p* < 0.05). Moreover, all these NFR parameters are comparable to the Sham group of rats (Figure 5b).

### 3.3. Histological Assessment

**Figure 6:**
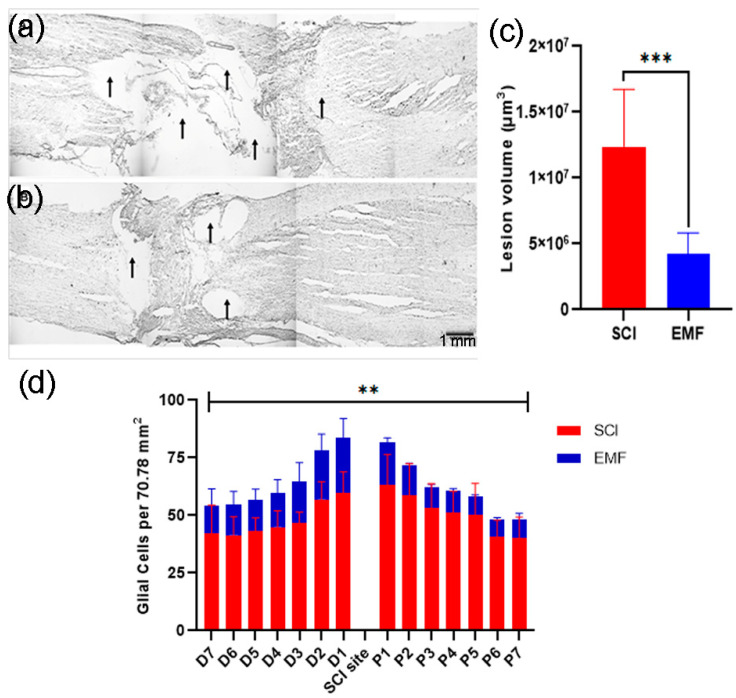
Neuroprotective effect of EMF after complete SCI. The representative spinal cord images of SCI (**a**) and EMF (**b**) groups. Scale bar = 1 mm. Arrows show the lesion cavity in both groups. Lesion volume shows a comparison between SCI (*n* = 5) and EMF (*n* = 6) groups of rats (**c**). The graph shows the number of glial cells per field at different locations from distal (D1–D7) to proximal (P1-P7) including injury in the six alternate longitudinal spinal cord sections of SCI (*n* = 3) and EMF (**d**), *n* = 3. *** *p* < 0.001 indicates a comparison between SCI and SCI-treated EMF groups. A *p* < 0.01 (**) indicates a glia cell comparison between the SCI and SCI-treated EMF groups at all points (proximal-distal).

Rats were sacrificed and perfused with paraformaldehyde for histological studies. The spinal cord histology in cresyl-violet-stained slides was studied for lesion volume in each group of rats (Figure 6a,b). It was found that the lesion volume was significantly higher (*p* < 0.001) in the SCI spinal cord at week 8 of SCI as compared to EMF, which suggests the protective role of EMF post-SCI (Figure 6c). For glial cells (astrocytes) counting, cresyl-violet-stained sections (6/rat) were studied on either side of injury (proximal/distal, Figure 6d). Briefly, we identified the astrocytes, which were larger than the microglia but smaller than the neurons, possessing pale, glassy nucleus, and cytoplasm. For these cells, the nuclear envelope was sharply outlined by a thin chromatin rim. The data were analyzed and compared between the SCI and EMF groups in each frame (70.78 mm^2^) of caudal and rostral from the injury center.

The number of glial cells was higher adjacent to the injury site. In all the frames (both sites), the total studied number of the glial cells was more in SCI (*p* < 0.01) as compared to EMF, further suggesting a beneficial and protected role of EMF stimulation after SCI.

### 3.4. Neurochemical Assessment

To study the beneficial effect of EMF stimulation on the injured spinal cord, we parallelly study the effect of EMF on glutamate concentration throughout the spinal cord including the injury site. Therefore, different regions of the spinal cord were dissected and analyzed for glutamate concentration using the LC-MS-ESI system (Table 1). We found no statistically significant change in the cervical, thoracic, lumbar, and sacral regions of the spinal cord in all the groups, though the level was elevated in the SCI and EMF groups. Moreover, glutamate concentration was higher even after 8 weeks of SCI at the site of injury (*p* = 0.001) only as compared to Sham; whereas, it was restored (*p* = 0.04 versus SCI) and comparable to Sham by everyday EMF stimulation for 8 weeks (Table 1).

## 4. Discussion

The study demonstrates progressive, accelerated, and heightened sensorimotor and electrophysiological responses to noxious and non-noxious stimuli and an increase in glutamate concentration at the SCI site with a concomitant lesion volume in adult male rats after complete thoracic SCI. Daily (2 h/day) EMF stimulation (50 Hz, 17.96 μT) significantly reversed these effects, which were significant at week 6 after SCI. The data suggest a beneficial effect of EMF stimulation on SCI-induced sensorimotor deficits including allodynia and hyperalgesia, probably by attenuating the excitotoxic effect of glutamate. Besides, it provides the temporal pattern of nociceptive responses to a variety of noxious stimuli, correlation with related electrophysiological parameters such as H-reflex, NFR, and glutamate at the injury site to explain the beneficial influence of EMF stimulation in the same rat.

The exaggerated motor responses of the tail to non-noxious stimulation (10 °C) indicate allodynia, while noxious hot and cold stimulation indicate hyperalgesia, which is in line with the literature. The present study suggests a characteristic temporal pattern of allodynia and hyperalgesia, which is progressive. These responses are attributed to progressive secondary injury of the spinal cord including hyperexcitability of spinal sensory neurons [17,18,19]. It is very well known that electromagnetic stimulation (EMS) noninvasively can induce electric current from the surface to underlying tissue [20] and generate locomotor responses in non-injured humans and cats [21,22]; additionally, there are recent reports of beneficial effects of variable EMS in different SCI animal models [23,24,25,26] besides our laboratory recent report [27]. Bhattacharyya et al. reported that daily EMF stimulation (17.96 µT, 50 Hz, 2 h/day for 3 weeks) of contusion SCI rats reduced the voltage-gated calcium channel expression in the spinal cord, which is the main cause of glutamate-associated excitotoxicity in SCI animals as well as the hyperexcitability of spinal sensory neurons [27]. This is also associated with the neuroprotective and neuroregenerative effect of magnetic stimulation after SCI [25,26,27]. Liu et al. found that daily repetitive trans-spinal magnetic stimulation (rTSMS; 5 Hz, 1.5 T, 10 sequences of 20 s for 8 weeks) enhances the locomotion and associated expression of 5-HT and GAP-43 in the injured spinal cord, as well as reducing the apoptosis [26]. Similarly, another mechanistic study by Chalfouh 2020 supported our outcomes. They found that rTSMS stimulation (10 Hz, 10 min/day for 14 days, 10 s ON/20 s OFF of 0.4 T) is beneficial in reducing glial scar/inflammation and promoting neuroregeneration/survival, thereby enhancing locomotion using different lesion paradigms and types in the wild-type as well as the transgenic animals at different ages such as juvenile, adult, and aged [25]. In addition, it modulates the different neuroregenerative growth factors, which are also found in our previous study [25,28].

In the primary spinal cord injured segment of SCI rats, a significant increase in glutamate concentration was obtained, which is in line with a 37-fold increase reported after contusion SCI [29,30,31]. It activates glutamate receptor-mediated critical biochemical pathways leading to oxidative stress, irreversible central sensitization, dysfunction of the glutamate transporter, and reduction in protein kinase C activity [18,32]. In a normal rat, a high-affinity, sodium-dependent glutamate transporter is predominantly expressed in astrocytes, which accounta for >80% of the total glutamate uptake in the brain from extracellular space [33]. The glutamate concentration was significantly attenuated by EMF stimulation in our rats, which again contributes towards a significant containment of secondary injury involving free radical generation, inflammation, excitotoxicity, and hyperexcitability, as suggested by others using different types of magnetic stimulation [34]. Another study reported the role of EMS on the modulation of NMDA receptors related to glutamatergic synaptic plasticity in lumbar motoneurons after SCI [34]. They found that EMS (0.2 Hz for 35 min) on the spinal cord promotes transmission and facilitates NMDA receptor function including at motoneuron synaptic inputs from lateral white matter and corticospinal tract after hemisection SCI in rats without [34] or with exercise in combination [35]. It was also suggested that EMS not only modulates the lumbar motorneurons’ plasticity but also strengthens the neuro-muscular circuits and spinal networks after SCI in humans and rats [36].

Nevertheless, there is sufficient evidence to believe that hyperalgesia is primarily dictated by hyperexcitability of spinal neurons [37], which is also reflected in our H-reflex study in the same rats. These results are in line with the previous reports of alteration in muscle and nerve compound action potentials after SCI [38]. This is attributed to an alteration in axonal excitability and conduction properties, secondarily to a shift in the regulation of intracellular and extracellular ion contents or changes in the neuronal expression of Na+ and K+ channels [38,39]. Additionally, a marked decrease in the number of motor neurons due to injury per se and reduction in motor unit size due to disuse atrophy of muscles are implied in the decrement of M-response amplitude after SCI [24]. Since the amplitude of M-response was restored in our EMF-stimulated SCI rats (Figure 4), it is pertinent to submit that disuse atrophy of the muscles may have been significantly attenuated in them, probably secondary to the improvement in their BBB score [9,24]. They have also shown the improvement in locomotor behavior and muscle physiology by pulsed EMF in the SCI mice. We further substantiated the beneficial effect of EMF on SCI-induced hyperalgesia and allodynia by including objective evaluation via recording polysynaptic NFR in the same rats (Figure 5).

As documented in the literature, the threshold and latency of NFR in our SCI rats also increased, while the duration and amplitude of it decreased, which indicates post-SCI hypoalgesia in sharp contrast to their behavioral hyperalgesia. The controversy is probably either due to the design of the experiment involving an anesthetized rat or the recent insult to the reflex center, since we recorded the response from the biceps’ femoris after stimulation of the sural nerve. The polysynaptic neural circuits and the recently injured reflex center of NFR are both susceptible to anesthesia. In contrast to SCI rats, the NFR parameters of our EMF-stimulated SCI rats were comparable to Sham, thereby reiterating the previous conclusion of the beneficial effect of EMF. Therefore, our behavioral and electrophysiology studies suggest the predominant contribution of hyperexcitability of spinal neurons in post-SCI pain, secondary to an increase in glutamate, which was restored by EMF stimulation. While, hyperexcitability and pain also contributed significantly to the microglia cells, which send a signal through prostaglandin E2 to spinal dorsal horn neurons and abnormal sensory processing [40]. EMF stimulation in our SCI rats restored eualgesia by its multifactorial action involving all these processes.

It is well known that inflammatory response and progressive necrosis after SCI increase proximally and distally (15–20 mm) from the initial focus. In the nervous system, microglia are the principal phagocytes mobilized and activated. It is activated via the local release of factors such as interleukin 6, fractalkine, and cysteine–cysteine chemokine ligand-2. Besides hypertrophy, the activated state is characterized by increased production of cell-specific markers, pro-inflammatory cytokines, reactive oxygen species, ATP, excitatory amino acids, and nitric oxide. All these significantly contribute to secondary damage after SCI. In addition to inflammation, secondary damage includes ischemia, free radical release, apoptosis, and excitotoxicity [18,41]. These processes are reduced or restricted by magnetic stimulation after SCI, as shown in the literature, and thereby, the functional recovery after SCI in our rats.

On the other hand, EMF stimulation has been shown to promote angiogenesis and blood supply, reduce oxidative stress, improve wound healing, and attenuate apoptosis in general [42,43,44,45] as well as in SCI animal models. The present study provides robust evidence for elevated levels of the excitotoxic neurotransmitter and preliminary evidence of inflammatory response after SCI week 8; whereas, EMF stimulation in the SCI rats substantially attenuated this process by reducing the glutamate associate neurotoxicity. The reversal in glutamate concentration supports the improvement in sensorimotor behavioral deficits and receives strength from its electrophysiological correlates, as supported by other reports. In support of attenuation of the inflammatory response in our EMF-stimulated rats, we report for the first time a reduction in the glial population after SCI. Attenuation of microglia reaction and activation besides containing inflammation also contributes significantly to check the post-SCI hyperexcitability of sensory neurons and thereby the pain behavior. The hyperalgesia was notably recovered after week 4, and allodynia was surprisingly not seen in our EMF-stimulated rats. A recent report suggests that EMF stimulation improved the SCI-induced spasticity and gait impairment via the up-regulation of dopamine beta-hydroxylase, glutamic acid decarboxylase 67, gamma-aminobutyric acid B receptor, and brain-derived neurotrophic factor in the lumbar spinal cord segments [46].

The underlying mechanism for restoration by EMF possibly also includes opioids besides NMDA receptors modulation [34,35,36], since EMF significantly relieves the pain of a wide variety via μ and δ opioid receptors [47] and increases the endogenous opioid and serotonin levels in the hypothalamus as well as serotonin in the brain stem [48]. Our study suggests that EMF stimulation attenuates hyperalgesia and allodynia in rats with complete thoracic SCI. The recovery was statistically valid after SCI week 4, and the study gains support from the attenuation of lesion volume, glial cell response, and concentrations of glutamate.

## 5. Conclusions

Our study suggests that EMF stimulation attenuates hyperalgesia and allodynia in rats with complete thoracic SCI. The recovery was statistically valid after SCI week 4, and the study gains support from the attenuation of hyperalgesia/allodynia, lesion volume, glial cell response, and concentrations of glutamate. It is a very useful and non-invasive SCI patient-friendly tool that can be used regularly in SCI clinics for patients’ welfare and their rehabilitation.

## Figures and Tables

**Table 1:** Effect of electromagnetic stimulation on glutamate after 8-week complete SCI.

Spinal Cord Regions	Glutamate Concentration * (Median with Range)			*p*-Value		
	Sham Group (*n* = 8)	SCI Group (*n* = 6)	EMF Group (*n* = 7)	Sham vs. SCI	SCI vs. EMF	Sham vs. SCI
Cervical	20,045.9 (933.3–25,762.7)	13,607.43 (3000–31,354.2)	18,198.42 (4907.4–70,602.4)	1.000	1.000	1.000
Thoracic	10,480 (2112.6–26,250)	19,575.1 (8262.3–67,272.7)	18,198.42 (4907.4–70,602.4)	0.058	1.000	0.378
Injury	5989.58 (90–23,760)	61,958.3 (11,303.0–173,333.3)	12,314.3 (2884.2–61,600)	0.001	0.044	0.644
Lumbar	16,297.2 (6785.7–23,418.8)	20,542.1 (3633.3–46,800)	22,810.6 (19075.6–30,731.7)	1.000	1.000	0.324
Sacral	15,271.1 (6735.6–26,753.2)	13277.1 (3571.4–43,846.1)	24,714.8 (21,212.1–31,159.4)	1.000	0.185	0.319

* Glutamate concentration (ng/g wet tissue).

## Data Availability

Data available on request.

## Supplementary Materials

The following are available online at https://www.mdpi.com/article/10.3390/brainsci11111431/s1, Figure S1: Representative images of MRI of the whole rat (*n* = 3) showing the intact pre-SCI (left, white arrow) spinal cord and injured post-SCI spinal cord (right side, white arrow). BBB score was recorded for these rats before MRI. Figure S2: Representation of rats used in each group. These rats as mentioned in the schematic successfully completed the study and all the behavioral tests including BBB score and sensorimotor tests (Sham; *n* = 15, SCI; *n* = 12, EMF; *n* = 14). These animals at week 8 were divided for separate studies such as electrophysiology and neurotransmitter, while another half for histological studies as mentioned in each figure legend. Figure S3: Schematic of testing rats for a battery of tests. All these rats successfully completed the behavioral (B, T) tests (Sham; *n* = 15, SCI; *n* = 12, EMF; *n* = 14) during the study period. At week 8, half of the rats in each group studied for electrophysiology and neurotransmitter studies (E/N), while another half for histological studies (H). B—BBB score; T—tail flick latency/threshold of tail flick.
