# Supplementary material for: Electromagnetic Field Stimulation Attenuates Phasic Nociception after Complete Spinal Cord Injury in Rats
Source: Brain Sci. 2021 Oct 28;11(11):1431. doi: 10.3390/brainsci11111431 (PMC8615391; doi:10.3390/brainsci11111431)
Supplement: Supplementary file 1 [file brainsci-11-01431-s001.zip › brainsci-1409204-SI.pdf]

brain sciences

Supplementary Materials

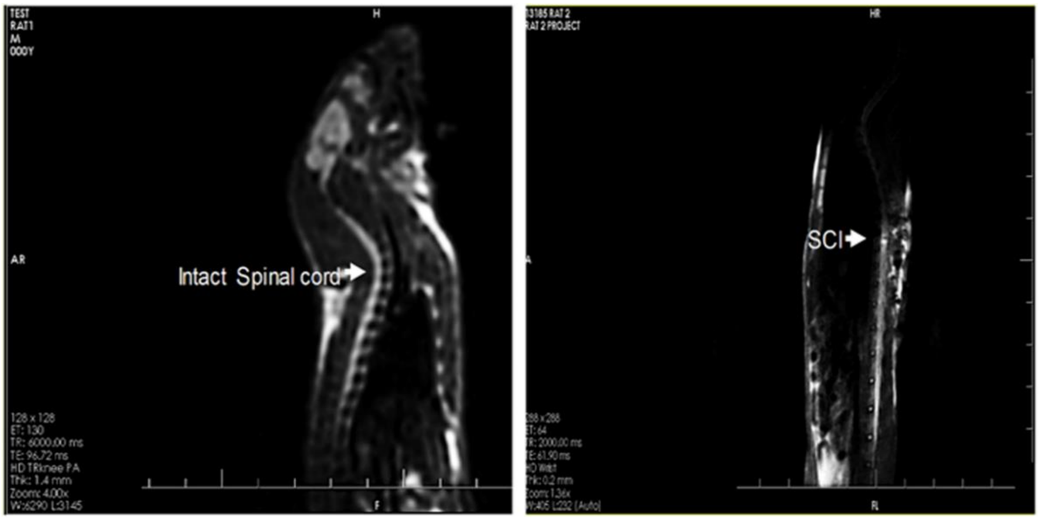

Figure S1: Representative images of MRI of the whole rat (*n* = 3) showing the intact pre-SCI (left, white arrow) spinal cord and injured post-SCI spinal cord (right side, white arrow). BBB score was recorded for these rats before MRI.

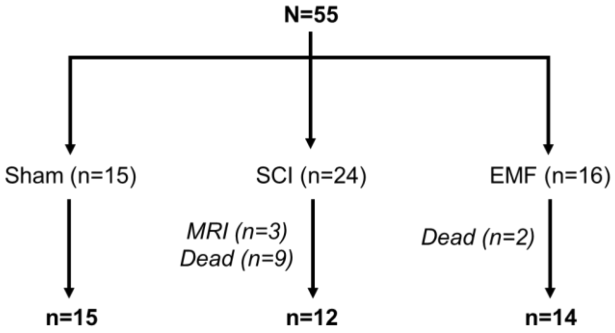

Figure S2: Representation of rats used in each group. These rats as mentioned in the schematic successfully completed the study and all the behavioral tests including BBB score and sensorimotor tests (Sham; *n* = 15, SCI; *n* = 12, EMF; *n* = 14). These animals at week 8 were divided for separate studies such as electrophysiology and neurotransmitter, while another half for histological studies as mentioned in each figure legend.

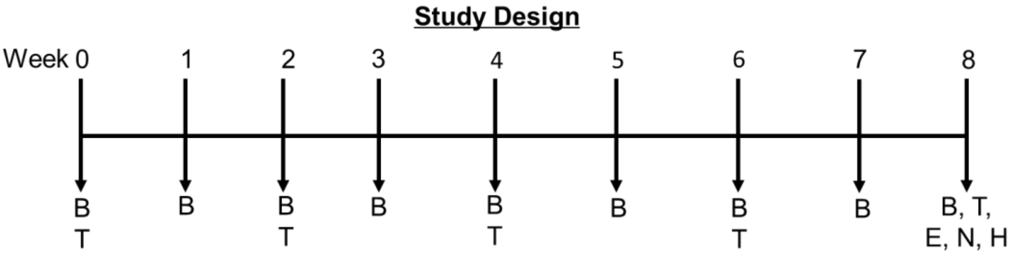

Figure S3: Schematic of testing rats for a battery of tests. All these rats successfully completed the behavioral (B, T) tests (Sham; *n* = 15, SCI; *n* = 12, EMF; *n* = 14) during the study period. At week 8, half of the rats in each group studied for electrophysiology and neurotransmitter studies (E/N), while another half for histological studies (H). B—BBB score; T—tail flick latency/threshold of tail flick.
